# The Hospital. Nursing Section

**Published:** 1903-09-12

**Authors:** 


### The Hospital.

### Nursing Section.

Contributions for this Section of "The Hospital" should be addressed to the Editor, "The Hospital" Nursing Section, 28 & 29 Southampton Street, Strand, London, W.C.

No. 885.—Vol. XXXIV. *SATURDAY, SEPTEMBER* 12, 1903.

### Notes on News from the Nursing World.

### ARRIVALS FROM SOUTH AFRICA.

The s.s. *Nubia*, which arrived at Southampton on Saturday, had on board Sister M. L. de Montmorency, of the Army Nursing Service, on leave for three months; and Sisters M. A. Bindloss, M. A. Goldby, and R. M. C. Carr, of the Army Nursing Service Reserve, who return in consequence of the reduction of establishment.

### THE CATHOLICITY OF NURSING.

It is of the first importance that the catholicity of nursing should be steadily maintained. At a recent meeting of the Bangor and Beaumaris guardians, when the question of the board's contribution to the Bethesda Nursing Institution was raised, two of the members alleged that it was "purely a Church of England Institution." This remark does not appear from the report in the local papers to have been either challenged or authoritatively confirmed. But we cannot for a moment believe that the institution has exposed itself to such a charge. If it had done so, the guardians would have been justified in withdrawing the financial support which it was decided, not only to continue, but to increase. Moreover, the Bethesda Association is affiliated to Queen Victoria's Jubilee Institute of Nurses, and it would be a violation of the fundamental principles of the Institute to set up anything in the nature of a religious test. One of the members who brought forward the accusation was asked by the chairman if he knew of any cases where assistance by the nurses had been refused, and no response was forthcoming. There are circumstances conceivable in which the help of a Queen's nurse might be justifiably withheld, but never on account of the particular religion, or irreligion, of the person requiring assistance. The avocation of nursing would be deprived of one of the great characteristics which entitle it to honour if it ceased to be as catholic as Christianity itself.

### THE FIRST AUSTRALIAN NURSES' CLUB.

Australian nurses are anxious to found a residential club for themselves, and the subject is already under discussion. Only fully-qualified nurses will be eligible, and the names of at least 100 suitable candidates desirous of joining must be received before any definite step can be taken. The originators of the scheme at Melbourne have wisely determined that the financial difficulty must be solved before the club can be built, and also that the house must be built specially, not adapted, in order to secure a sufficient number of small rooms. Members of the club will have to pay an entrance fee and a yearly subscription, so that country members can join and stay a the club when in town, paying for their board at the same rate as those always residing there. There is to be a lecture-room for meetings, etc., and a gradually increasing library, with newspapers, nursing journals, and magazines. Proper arrangements for members being nursed in their own rooms should they fall sick, will be made, and for disinfecting nurses who have been attending an infectious case prior to their joining the family circle. As to expenses, an attempt is to be made to provide for the nurses at comparatively small cost, good and wholesome food, with all the necessities, and some of the luxuries of life around them.

### NURSING IN IRISH WORKHOUSE INFIRMARIES.

The hope which is expressed in another column by a nurse in Ireland, that the Viceregal Commission appointed with the view of improving and reforming workhouses will not overlook the nursing question, has our entire sympathy. We trust that our correspondent, who occupies a position which should enable her to speak with authority, has had an exceptionally unpleasant experience and that the untrained woman to whom she refers is not so black as she is painted. But the Granard and other cases have conclusively shown that, although the Irish Local Government Board have manifested considerable energy in dealing with recalcitrant Guardians, there are still serious impediments to the progress of reforms in workhouse nursing, and one of these is undoubtedly the obstacles which are thrown in the way of the trained nurse wherever she is an innovation. Meanwhile, we can only recommend the practice of the grace of patience.

### GUARDIANS AND UNTRAINED MATRONS.

As might have been expected, the attempt to prevent the passing of the untrained workhouse matron is backed up by some of the Boards of Guardians, who cannot discern the signs of the times. There is no feeling against the many excellent individuals who have filled, and still fill, the position of workhouse matron because their education has been defective, and their knowledge of nursing is limited; but there is a very strong conviction that it is absolutely essential in the interests of the community that they should cease, as vacancies occur, to exercise power which they are not qualified to possess, and should, in due course, make way for officials who have had the advantage of a sound education, and are qualified by special training to see that the needs of the sick receive the attention they have a right to ask for at the hands of the State. We do not think that Mr. Long will be unwise enough to ignore this conviction in deference to the natural wishes of the class represented in the memorial of the masters and matrons, but in any event the ultimate reform of a doomed system cannot be delayed for a protracted period.

### STRANGE SITUATION AT A LONDON INFIRMARY.

At the end of last month Ladywell Workhouse Infirmary was for a short time left entirely to the mercy of the assistant master and the assistant matron. The matron and master were both absent, the head night nurse was away, and the superintendent nurse had departed upon the expiration of her time. In the night a death occurred among the females in the absence of the nurses, and the woman had to be taken care of by the attendants. The medical officer had done his best to prevent such a condition of things by asking that the superintendent nurse—who had resigned on account of her objection to have the medical book interfered with by the matron—should be asked to stay on until the committee had met and made other arrangements, impressing upon the Board the necessity of retaining the services of an official who thoroughly understood her duties. To that request the clerk had replied that a qualified trained nurse would be obtained, and that Guy's Nursing Institution had offered to send one. A nurse, however, did not arrive, and the fact was elicited at an extraordinary meeting of the Guardians that the assistant matron had agreed to temporarily act as superintendent nurse as well as do her own work, which called forth a remark from one of the Guardians that if such a thing were possible the building at Ladywell was overstaffed. Ultimately, the Board undertook to discuss the whole matter at their next meeting, and in the meantime engaged a temporary superintendent nurse at 22s. a week.

### NUNS AS NURSES.

The Guardians of the South Dublin Union were invited, at the instance of the chairman, to assent to a proposal for the appointment of five additional nuns as nurses and they have consented to make an increase of two, one of whom will serve in the children's infirmary and the other in the female Roman Catholic hospital. A Guardian, in objecting to any increase in the number of nuns, said that it was a practice for young nuns to come to learn something about nursing in the infirmary and leave at the end of six months to make way for more novices. But this was denied by other speakers, and Dr. Biggar, of the Local Government Board, supported the plea for the employment of more nuns. In the final resolution passed it was agreed that five qualified nurses should be employed for night duty.

### BICYCLES FOR DISTRICT NURSES.

Those who are brought much into contact with district nurses know well the weary miles they have sometimes to cover in the discharge of their duties. This fact has been forcibly impressed upon a lady living in Lancashire, who advertised that she had a bicycle to give away to a district nurse, and has in consequence received 40 applications from nurses living in scattered districts. These nurses have daily to visit numerous villages, and have mostly no means of conveyance except shank's pony. The lady with the bicycle now experiences a difficulty in choosing a recipient of it, while she confesses that her still harder task is to refuse the other urgent appeals. She thinks, however, that there are many owners of discarded bicycles which are still capable of good service, who would be glad to know of this really helpful way of getting rid of them. If so, we can assure them that there are district nurses, not only in Lancashire, but in all parts of the country, who would rejoice to have their labours among the poor lightened by the bestowal of such a useful gift.

### MASSAGE AND THE BLIND.

There are so few occupations which can be carried on by the blind that it is pleasant to learn that blind persons can very successfully learn and apply massage. In Japan, where massage is much in vogue, the blind man who is otherwise healthy can always earn a livelihood; and a notable feature of any Japanese town towards evening is the blind masseur as he walks along, announcing himself with his peculiar sounding whistle, in search of work, which he can always find in plenty. There is now a text-book in the Braille type which renders it possible for sightless pupils to materially assist themselves and their teacher during the course of instruction, and to refresh their memory when they become independent. Mrs. Creighton Hale's book "The Art of Massage," in Braille type, will help many afflicted people to take up useful and remunerative work.

### A PROBATIONER'S RESIGNATION.

The Bristol Board of Guardians at their last meeting spent upwards of an hour and a half in discussing the question whether a probationer had been fairly treated in being asked by the hospital committee to resign. It is very desirable that nurses in the employ of Guardians should be protected from injustice at the hands of a committee, but we cannot congratulate the Bristol Board on the fact that they were not able, even after prolonged debate, to come to a conclusion as to whether the Hospital Committee were competent to deal with the alleged grievance of a probationer. The case of the Committee was that, not being satisfied with the work of the probationer, they had requested her to send in her resignation, and the contention of the Guardians who raised the matter was that this course had been taken before the circumstances were properly investigated. The criticisms on the action of the Hospital Committee had previously resulted in the proposed resignation of several members, and it was urged that the resignations should be accepted. But as the voting in favour of the motion and against it was equal, and the chairman refused to give a casting vote, no decision was arrived at, and after some further wrangling the next business was proceeded with. It will strike most people, however, that unless the Guardians have sufficient confidence in their own Committee to determine whether a probationer should be required to resign, or whether her case should be disposed of by the Local Government Board, their continuance in office is a farce.

### The Examination of the Urine.

By J. K. WATSON, M.D.

### PART I.

The examination of the urine is not of necessity required of the nurse, but it is advisable that she should have an elementary knowledge of urine testing, since she can often put such knowledge to use and assist thereby the doctors under whom she is working. The subject, if considered in detail, is complex, and all that is now attempted is to discuss it in its more important and practical bearings. In the first place a few physiological and anatomical details will not be out of place. There is a constant wear and tear going on in our bodies, and this requires that waste material shall be removed. The organs concerned in this process are the kidneys, the skin, and the lungs. The chief waste materials of the body may be classified as follows:—(1) A substance called urea, which represents a large proportion of the food stuffs, which have been transformed into it in their passage through the body. (2) Carbonic acid, which leaves the body in the expired air. (3) Various salts, and (4) water. The urea is nearly all discharged by the kidneys. The salts are chiefly eliminated by the kidneys, to a slight degree by the skin, and the water leaves the body by all three channels, but chiefly by the kidneys.

It will be seen, then, that the kidneys are the most important organs for the removal of waste material, or, as we commonly say, excretory organs.

The urinary apparatus consists of the two kidneys, right and left, their respective ducts, the ureters, the bladder, or reservoir where the urine is stored, and the outlet of the bladder, the urethra, which ends in an aperture, the meatus. The kidneys lie on the posterior wall of the abdomen, in the loin, behind the peritoneum, which lines the abdominal cavity and covers, to a large extent, its contents. This is of importance to the surgeon who is able to reach the kidneys from behind without interfering with the peritoneum. Their shape is so well known that we are accustomed to use the term kidney-shaped as a descriptive one. They are surrounded by a varying quantity of fat. Their length is about four inches and they weigh about four and a-half ounces. The inner or concave edge of the organs contains a depression called the hilum. Here the ureter leaves the kidney and the blood-vessels and nerves pass into and out of the organ. The kidneys are covered with a tunic, the capsule, which, in the healthy organ, can be stripped off, but in disease of the kidney it may be difficult or impossible to remove it. The essential structure of the kidney, the secreting part, consists of a complicated system of tubules and tufts. The former communicate with a cavity, called the pelvis (Latin pelvis, a basin) of the kidney, which gives origin to the ureter. This cavity may indeed be regarded as the upper, swollen, or dilated end of the ureter.

Sometimes it becomes enormously distended, at the expense of the kidney substance. Such a condition is liable to occur when the ureter becomes blocked, as may occasionally happen from the impaction of a stone (calculus) in its interior, and the urine is dammed back in the kidney. The urine which is poured out by the tubules is collected in the pelvis of the kidney, to be discharged by the ureter into the bladder. The ureters pass down obliquely to enter the back wall of the bladder, one on each side, by a valvular opening. These openings are only about two inches apart, and are so arranged as to prevent a reflux of the urine. The ureters are about sixteen inches long and their diameter is that of a goose-quill. Their position is of great importance to the surgeon in connection with certain operations, especially on the uterus (womb), as they may be easily damaged. The bladder varies in shape according to its degree of distension; when moderately full it is rounded, but it becomes more ovoid in shape when it is fully distended. In the adult the bladder lies in the pelvis in front of the rectum and behind the pubic bones. In the female the uterus is placed between the bladder in front and the rectum behind. When the bladder becomes very full it rises up out of the pelvis, and may even reach up to the umbilicus in cases of retention of urine, where the urine, for one reason or another, cannot be passed. Such a condition as this (retention of urine) must be distinguished from suppression of urine, where there is no urine secreted at all. The bladder may be said to hold, in the adult, about a pint of urine, but its capacity varies very greatly. The urine passes down into the bladder partly by the action of gravity, and partly by muscular contractions taking place in the walls of the ureter, similar to those which take place in the walls of the intestine (peristalsis). It is evacuated by a complex mechanism, which is usually under our control, but under certain conditions may act involuntarily.

The urethra is situated at the lower and front part of the bladder, and differs in its anatomical relations in the two sexes.

### PART II.

Having now briefly described the urinary apparatus we next pass to a description of the urine, but before doing so it will be well to refer to the function of the kidneys. How do they produce the urine? The urine is secreted in virtue of two conditions, namely, a mechanical one and a vital or selective one. The tufts, to which we have referred, are collections of capillaries surrounded by a capsule, and these act largely as filters, through which the watery part of the urine, together with a small part of the solids passes under a varying degree of pressure. The more important part of the secretion of urine, however, is that which occurs in the tubules; for these have the power to select and to refuse the solid constituents of the urine. Thus their action becomes a vital one, and this selective action is performed by their lining membrane, which consists of a special (glandular) form of epithelium. This epithelium will be referred to later on when we come to speak of abnormal constituents of the urine. Suffice it to say that on its power of performing this selective task efficiently depends, in large measure, the healthy condition of the urine and of the body generally.

### Description of the Urine.

We shall describe the urine under certain heads, pointing out in each case some of the variations which are commonly met with both in health and disease:—

I. *Quantity.*—This varies with the quantity of liquid drunk, also with the temperature; for, when the skin acts freely, as it does in warm weather, less water leaves the body by the kidneys and vice versâ. The average quantity passed in the 24 hours is 50 ounces, but the limits in health may be said to range from 35 to 60 ounces. Less urine is usually secreted during sleep. Children, of course, pass less urine than adults, but after the age of 15 the amount passed is much the same as in adults. Nurses should, in estimating the quantity of urine passed in the 24 hours, keep a separate record of the "day urine" and "night urine" and add these together to get the sum for the 24 hours.

When the "night urine" approaches to or equals in quantity the "day urine" an abnormal state of affairs exists,

THE EXAMINATION OF THE URINE—*Continued.*

and such a symptom calls for investigation. For instance, this may be an early sign of chronic kidney disease. It is often difficult or impossible to collect all the urine voided, especially in children, and in cases where great accuracy is of importance a catheter must be used.

In the various fevers the urine is diminished in quantity, also in acute inflammation of the kidneys (acute nephritis) and in cases characterised by profuse diarrhœa or perspiration where most of the water leaves the body by the bowels and the skin respectively. Heart disease, too, may cause a diminution in the quantity by lowering the pressure under which the urine is secreted. In cases of great prostration and collapse, such as occur in cholera and severe head injuries, the kidneys may fail to secrete any urine (suppression of urine). The same may occur, too, in long standing kidney disease where both kidneys are extensively diseased.

The quantity is increased in the two forms of diabetes—the one form (diabetes insipidus) being characterised merely by the regular passage of a very large quantity of urine in the 24 hours, and the other (diabetes mellitus) by, in addition, a varying amount of sugar in the urine. The earlier stages of chronic kidney disease cause an increased quantity, and some diseases of the nervous system, notably hysteria. Drugs which act more especially in promoting a flow of urine (diuretics) have also the same effect.

II. *Colour.*—This is due chiefly to two pigments, urochrome and urobilin, especially the former. It varies from pale yellow to darkish brown, even in health, and we may thus classify urines as to their colour, as (*a*) pale; (*b*) normal coloured, and (*c*) high coloured. As a rule, the greater the amount of urine passed the paler is the colour and the smaller is the amount of solids in solution, and vice versâ; thus we are accustomed to look for pale urine in cases of diabetes and hysteria and for high coloured urine in fever The urine may be altered in colour from the presence of bile, as in the case of jaundice, blood and various pigments other than those we have mentioned. Several drugs, too, colour the urine; for instance, carbolic acid, which produces an olive-green tint, santonin and rhubarb which increase the reddish tint.

III. *Transparency.*—Healthy urine is nearly always clear when passed, but soon a cloud of mucous forms which causes a slight turbidity. The transparency may be impaired or lost in various conditions as when the urine contains mucus (excess of), phosphates, urates, blood, pus or albumin. The presence of oxalate of lime (oxaluria), too, causes a cloud in the upper part of the urine glass. The urine may thus be turbid under a variety of conditions.

IV. *Specific Gravity.*—By this, which is usually written as "sp. gr.," is meant the ratio between the weight of a given volume of urine and an equal volume of water, the latter being regarded as 1,000. It depends on the amount of solid matter in the urine, and varies from 1,015, or even less, to 1025 in health. The average is 1020. The specific gravity is taken by means of a urinometer, a glass apparatus weighted by mercury and having a graduated stem. Its use we shall describe when we speak of urine testing. Before drawing any conclusions from the specific gravity, we should ascertain the quantity of urine passed. In pale watery urine the specific gravity is generally low; when it is high in such a urine it is generally due to the presence of sugar. In concentrated high-coloured urine, the specific gravity is high from the large quantity of solids present. Albuminous urine has usually a low sp. gr.

*(To be continued.)*

### Nurses and Dispensing.

### LONDON SCHOOLS OF PHARMACY.

(*Continued from Page* 290.)

### THE METROPOLITAN COLLEGE OF PHARMACY.

This college was established in 1893 "with the object of providing the pharmaceutical student with education in those branches of science which are directly related to pharmacy, and to complete the training in practical pharmacy partially acquired in business during the term of apprenticeship. . . . The aim is to equip students for business as well as for examination."

Students are prepared for the examinations of the Pharmaceutical Society and for the Assistants' examination of Apothecaries' Hall; the majority, however, take the former. The fees for the minor course are: Winter session (September 1st to December 31st), £10 10s.; spring session (January 1st to April 14th), £10 10s.; summer session (April 12th to July 23rd), £10 10s.; spring and summer sessions (January 1st to July 23rd), £19 19s.; tutorial course (September 1st to October 12th), £6 6s.; summer session and tutorial course (April 12th to October 12th), £15 15s.; full college course of three sessions, £28 10s.; half term, £6 6s.; per week, £1 1s. These fees are inclusive, and are payable on entry. Special fees can be arranged for students taking up any particular branch of work. The apparatus required for practical chemistry, which the student must provide himself with, costs £1 1s. For the Apothecaries' Hall course the fees are: for one session, £6 6s.; two sessions (full course required by the authorities for qualification), £11 11s. Arrangements can be made for candidates to take special subjects or part of a course. The Principal is Mr. W. Watson-Will, Ph.Ch., F.L.S., F.C.S., who is assisted by three demonstrators who have been pupils in the college. The buildings consist of two houses—Nos. 160 and 162 Kennington Park Road, S.E.—a few minutes' walk from Kennington Oval. For some years the work was carried on in one house only; the one adjoining has, however, recently been acquired, and a laboratory and lecture room have been built on the garden space at the back. The chemical laboratory occupies the upper storey of the new building, and is 70 feet long, 21 feet wide, and 15 feet high. It is lighted from the roof, and has from 80 to 90 benches. Gas, water, and a set of reagents are supplied to each bench. There are fume cupboards at intervals. The old laboratory is used when necessary. The balance-room is close by the new laboratory, and has a tiled floor, so that vibration is avoided as far as possible. Other rooms are a pharmacal laboratory, a dispensary, materia medica and pharmacy museums, histological laboratories, physics rooms, prescription-reading room (where there is a collection of nearly 4,000 autograph prescriptions), and a distillation-room provided with retorts, condensers, receivers, combustion furnace, and provision against fire. The museums contain a large number of specimens, some of which have been sent home from abroad by former students. In the lecture-room already alluded to there is an electrical optical lantern for illustrating botanical and other lectures. It is a light and airy room, corresponding in size and shape with the laboratory above, and it is lined and seated in pitch-pine. There are

NURSES AND DISPENSING—*Continued.*

two offices—one occupied by the Principal and the other by the secretary and demonstrators—and there is ample cloak and bicycle accommodation. In all there are 20 rooms in the two houses; and the one house being exactly like the other, there is a double set of class-rooms, museums, etc., the idea being that a small number of students may work in each and not inconvenience one another by overcrowding. There are three sessions—winter, spring, and summer—and a special tutorial class from September 1st to October 12th to prepare for the October examination. At the end of each session there is a college examination in practical and theoretical work, in which students who have attended 80 per cent. of the lectures and classes may compete, and one silver and five bronze medals are awarded, as well as certificates of merit. There are evening classes from 7 to 9 P.M. for students engaged in business during the day. The fees per session are:—Chemistry, physics and pharmacy, £1 1s.; practical pharmacy and dispensing, £1 11s. 6d.; analysis, £1 11s. 6d.; materia medica, botany and Latin, £1 1s.; entire course, £4 14s. 6d. Ladies are admitted to the college.

There are athletic clubs for students, and an annua dinner takes place.

THE LONDON COLLEGE OF CHEMISTRY, PHARMACY, AND BOTANY.

This college, "established for the purpose of providing thorough and practical instruction in all branches of science, especially for students preparing for Pharmaceutiacal, Medical, Apothecaries' Hall, and University examinations," is at 323 Clapham Road, S.W. The Principal is Mr. Henry Wootton, B.Sc.Lon., and he is assisted by qualified demonstrators.

The college, formerly a ladies' school, is a solidly built private house, perhaps a hundred years old. It stands back from the wood-paved road by about 60 feet of gravel drive, and the house and garden occupy a third of an acre. The rooms have been appropriated to use as chemical, pharmaceutical, and physical laboratories, lecture hall, museum, and class-rooms. All are fitted with electric light, and are well ventilated. They are heated by gas stoves standing out a little way, with flues, and at the time of inspection all was fresh and clean. The two large chemical laboratories are on the ground floor, and each student has a separate bench fitted with gas, water, and sink, the last being made after Mr. Wootton's own plan. The sink, which is of porcelain, is let into the bench between two students, and each sink is provided with two taps. The waste water is carried away in an open trough with the object of preventing stoppages, and in order that the troughs may be accessible for cleaning at any time. Each bench is provided with a locker. The lecture hall is capable of seating 65 students, whose desks are numbered. There are five large windows, and by means of a double set of blinds the room can be easily darkened for the electric arc lantern used in lectures. There is a sliding double blackboard, and gas and water are laid on for the operator for use in demonstrations.

The balance-room is on the ground floor, and easily accessible from the laboratories; the windows look out on the garden. The dried plants required to be recognised in the Minor examination are hung on the walls, and there are some German flower models in cases. A reference library is kept in this room. Adjoining the dispensary is a room set apart for distillations, nitrometer determinations, etc., which every student is required to perform. The materia medica and chemical specimens are arranged in glass cases and stoppered bottles, and conveniently placed for use. A catalogue is given to each student on entering the college. At the back of the house is a garden comprising about a quarter of an acre of land. This is used for growing the botanical and histological specimens required in the course of study. It is also a pleasant place in which students may sit and read or spend their moments of recreation. There is a roomy bicycle house.

There are three terms, beginning in October, January, and April, and in addition there is a special revision course of classes and practical work in all subjects, beginning the third week in August, for advanced students intending to enter for the October Minor examination. The course of instruction comprises lectures, morning and afternoon, on chemistry, physics, botany, materia medica and pharmacy, and practical work in the laboratories from eleven to five. A "rota" of work is hung in the entrance, so that each student may know his subject and room. In addition to lectures and practical work, tutorial classes are held daily, and each student attends not less than four during the week. The fees, inclusive of all lectures, classes, and practical work, are:—For the Minor Examination, one term, £9 19s. 6d.; two terms, £17 17s. For the Major Examination, one term, £6 16s. 6d.; two terms, £11 11s. For the Apothecaries' Hall Examination, one term, £5 15s. 6d. The fee for the Revision course is £5 5s.

Ladies are admitted, and there are evening classes on Mondays, Wednesdays, and Thursdays, from 7 to 9 P.M. The fees are:—For one evening per week, for three months, £1 1s.; two evenings per week, for three months, £1 15s.; three evenings per week, for three months, £2 7s. 6d. Special evening classes for the Apothecaries' Hall Examination, are held on Mondays and Thursdays. There are athletic clubs for students.

(*To be continued.*)

### Nursing Typhoid Fever in the East.

BY A CORRESPONDENT.

It may be interesting to nurses at home to hear something about the nursing of typhoid fever in the East. The fever itself varies in some ways from our English type, since in most cases it is complicated with malarial fever. This causes the temperature to run a very irregular course, sudden high temperatures being followed by equally sudden drops, and these again by further irregular rises, which is due to the malarial element present. The type is generally found in Greece, where the inhabitants are very subject to attacks of malarial fever, especially the soldiers, who come from the wilder inland districts, and from surrounding islands. These men are mostly rather undersized, miserable specimens of mankind as regards their physique, and their want of stamina shows itself very markedly when they have to fight against disease. A great point in their favour, however, is that they are not heavy drinkers, and consequently do not get such noisy and violent delirium as I have met with amongst British patients of the same class. The diagnosis of the malarial complication is made by microscopical examination of the blood for the malarial organism. Many of the cases of typhoid fever do not develop spots at all, but if they do, they are generally very profuse and marked. Nursing typhoid fever is very heavy, as it is customary for all patients with a temperature over 102 5° F. to have three-hourly baths day and night. This in a ward of, say, 36 beds, means incessant work for the nurses. The bath is given at tem-

NURSING TYPHOID FEVER IN THE EAST—*Continued.*

peratures varying from 33° C. to 37° C.—the patient remaining in 15 to 20 minutes—during which time cloths are put on the head and cold water constantly poured slowly over them. If the face gets very blue or the pulse bad the patient has small drinks of brandy given to him while still in the bath, or sometimes hot beef-tea with brandy in it. When taken out of the bath he is left wrapped in a sheet and blanket, the temperature being taken five minutes after leaving the water. Generally there is a drop of two degrees and sometimes very much more; in the latter case hot bottles are put to the feet and more brandy given, and if any sign of collapse is noted, a hypodermic injection of ether is administered. After remaining ten minutes wrapped up, the patient is taken out of the sheet and blanket, thoroughly dried and made comfortable, a blanket instead of a sheet being placed next the skin.

### The Value of Hypodermic Injections.

Collapse from heart-failure is very common, and in these cases I have seen wonderful recoveries occur from the use of hypodermic injections of camphorated oil given alternately with similar injections of strychnine, caffeine, or sparteine. The best way to give a camphorated oil injection is to put the needle straight into the buttock. If given in the usual way in the arm, it is very liable to make a deep sloughing abscess which takes a long time to heal and leaves a nasty scar. Given in the other way, it rarely, if ever, forms an abscess. Too much care cannot be taken when giving these hypodermics to make everything as aseptic as possible. Our instructions are always to make sure that the needle has been boiled since the previous time of use, and then to pass a 5 per cent. solution of carbolic acid through it several times. The part where the injection is to be made is well rubbed with ether, and the nurse's hands washed before touching the skin. In cases of collapse or great exhaustion, transfusion of normal salt solution is made every four hours, about a pint at a time being injected, generally into the abdomen or side of the chest, this, of course, the doctor always does. The diet ordered is generally milk and beef-tea and egg-flip, till the temperature has been normal five days; then an egg is given lightly boiled and soft bread and butter. If the temperature still keeps normal, the patients are very early allowed chicken and then full diet.

### Treatment of Convalescents.

Directly convalescence has set in they have two ounces of quinine and Kola wine three times daily, for two or three weeks; it is a very effectual tonic. Very little brandy is given during the fever, and only to cases in which great exhaustion is present. The principal complications of typhoid here are pneumonia, thrombosis, and erysipelas; peritonitis, perforation, or hæmorrhage are very rare. Cases with pneumonia undergo the same bath treatment, but between the baths, during the day, they frequently have dry or wet cupping followed by linseed and mustard poultices, and at night a cotton-wool jacket. If in these cases there is much consolidation of the lung oxygen is freely used, as well as hypodermics. For thrombosis the leg is wrapped in cotton-wool bandages and put in a padded wire splint.

### The Danger of Complications.

Erysipelas is a complication much to be dreaded, as it sets in quickly and generally death ensues rapidly, within a few hours. I remember one case especially. The patient had a very severe attack of typhoid, but was making a good recovery, when one afternoon at 4 P.M. I noticed that the right side of the chest looked very red and puffy. It looked very much like the effect of a poisonous sting, but I could find no mark of one. Applications of lead lotion were made, but the swelling increased and spread right across the chest rapidly. Towards 8 P.M. the patient began to wander in his talk, his face got cyanosed, his pulse feeble and rapid, and he expired at 11 P.M. In another case the erysipelas attacked the left side of the face and head, the patient recovered, but had very offensive discharge from the ear for weeks afterwards. In hæmorrhage the baths are stopped and ice bags applied to the abdomen, but these cases are very rare out here. Most of the cases treated by baths suffer from very persistent deafness for some time after they are quite well, and are very prostrate and exhausted during the convalescence. The three-hourly bath seems to exhaust the patients very much, since they have little chance of rest or sleep as there are so many things to be got in between whiles. The baths thrice daily, and none at night seems to be quite as effectual and does not make the patients nearly so exhausted.

### Everybody's Opinion.

[Correspondence on all subjects is invited, but we cannot in any way be responsible for the opinions expressed by our correspondents. No communication can be entertained if the name and address of the correspondent are not given as a guarantee of good faith, but not necessarily for publication. All correspondents should write on one side of the paper only.]

### A WARNING.

Miss C. E. Jones, matron, writes from Blenheim House, 22 Wilkinson Street, Sheffield: A few weeks ago I advertised in The Hospital that I had a nurse's cloak for sale, and amongst the applications I had for it was one from a Nurse Muriel Watkins, who described herself as doing private nursing on her own account at 3 Harvie Street, Paisley Road, Glasgow, and who also enclosed a card asking if I would send the cloak on approval. This I did, fully expecting that I was sending it to a nurse whose character I never for a minute doubted. Now I find she has left the lodgings in Glasgow, taking the cloak with her; and the police, both here and at Glasgow, seem to have lost trace of her. I thought I would like to warn any nurses of such a person, and if you care to insert it in The Hospital you may do so. I think it is a disgraceful thing for any nurse to do, and if I could I should very much like to bring the person to justice.

[The moral of our correspondent's experience is obvious. Articles should not be sent on approval to strangers.—Editor, The Hospital.]

### NURSING IN IRISH WORKHOUSES.

"Trained Nurse" writes from Ireland: It is to be hoped that the Viceregal Commission appointed to improve and reform workhouses in Ireland will not overlook the nursing question. Since the introduction of trained nurses into these institutions many desirable changes both in the management of the hospitals and the efficient care of the sick poor have taken place. But at what cost? A nurse finds herself confronted by unceasing hard work. Perhaps sixty or seventy cases, all needing less or more attention. And her help? That of an untrained woman who has been installed years previously and who bitterly resents the appearance of the newcomer. None but those who have experienced it can realise the difficulties which surround a trained nurse owing to the attitude taken up by this untrained and uneducated woman. Seeing that her authority is no longer supreme she is determined to die hard. She incites the patients to distrust their "new nurse," to whom as often as not she alludes by some nickname. She bribes the attendants and ward servants to disobey her and treat her disrespectfully. In fine, she does everything her narrow intellect suggests to render the work of the skilled nurse fruitless, painful, and disheartening.

ANSWERING ADVERTISEMENTS.

"I. B." writes: I beg to call your attention to the following facts, and shall be glad if you can give them space in your paper. I have lately had occasion to answer many of the advertisements in The Hospital, and have sent, with copies of testimonials and photo, a stamped addressed envelope for reply, this having always been my custom, and, in my opinion, only fair to the people who may have many applications to answer. Not more than a year ago, I think, some remarks were made in The Hospital *Nursing Section* pointing out to nurses the necessity of doing this, and stating the inconvenience caused when it was neglected. Now I should like to give my experiences from the other side of the question. Being anxious, for private reasons, for an immediate case, I have recently answered both likely and unlikely advertisements, and I naturally thought that, having enclosed a stamped envelope, the least I could expect was a reply. However, several have been sent back with copies and photo, but nothing to say where they came from, the letter or two not obliterated of the post mark being my only guide. Two had written inside the flap "Suited," but gave no address. One did answer, but on a scrap of waste paper torn apparently from another letter. Worst of all, several have not even returned testimonials, photo, or stamped envelope, with the result that out of two dozen photos in uniform only about six remain in my possession, not to mention the loss in postage stamps. Comment is hardly necessary, but even supposing I was utterly unsuitable in every respect, one would imagine that common decency or self-respect might have suggested different treatment to this. Under the circumstances I suppose my only consolation must be to thank my lucky stars I was not the appointed "*one*" in any of the situations, and I am trying to live up to that idea! My sister lately had similar "experiences," and it would no doubt be of great general interest to hear what other nurses have to say on the subject.

NUNS AS NURSES.

"Merciful" writes: I have never worked with a nun, but with nurses for nearly seven years, and I would like to know what authority "Cecilia" has for terming us "one of the most irreligious body of women?" I can only think her experience of life is very small. What is religion? Surely a fairly substantial total is—Do thy duty, and love thy neighbour, as thoroughly and conscientiously as possible. Let us not profess and call ourselves Christians. Let us rather glorify our God in doing the daily round and common task—doing cheerfully each duty lying nearest, small or great, pleasant or otherwise! I do not doubt the hardest lot is borne by our patients (in many cases), but God knows a nurse's life is sufficiently hard and depressing without anyone, by ungenerous judgment, making it harder. To those who condemn us, I say with a late poet—

"Possibly, too, seen in God's perfect sight,
Some lives we deemed black 'gainst our own may look white."

"Cecilia" writes: "Trained Nurse" undertakes to criticise the nun professionally, but her whole case turns on a matter of opinion. She quotes Mr. Wyndham as saying that "the nuns ought not to be asked to perform some parts of hospital work." If this is so where have they failed in their duty? As she is a "trained nurse" she must surely know that in hospitals worked by professional nurses there is a great diversity of opinion as to how far a nurse's duty extends, and consequently the practice of different hospitals varies considerably, so surely the nuns are justified in protesting against a routine which is not obligatory on many professional nurses. It is a curious method by which "Trained Nurse" proves that what the nuns declined to do was part of their duty. She argues that the nuns ought to have done it because other people were found who would do it. It is difficult to see that everyone is bound to do whatever any other person can be made to do, and is it not possible in this case that nurses have too readily undertaken what was neither their duty nor the duty of the nuns, but that of some third person? "Trained Nurse" takes exception to nurses being called, as a body, irreligious. Is it not rather the cause for the deepest regret that it is too true, and are not the matrons of our large hospitals ever trying to instil into probationers the high moral tone that should belong to our noble profession. Fortunately there are many nurses, as well as nuns, upright sympathetic women, leading holy lives, but looking on nurses as a body, and nuns as a body, how do they compare for instance in the presence of death? in the presence of an unconscious patient? during a post mortem examination? or in conversing and comparing their cases? There seems something still to learn from the nuns who taught us the very necessity of our existence.

### Appointments.

[No charge is made for announcements under this head, and we are always glad to receive, and publish, appointments. The information to insure accuracy should be sent from the nurses themselves, and we cannot undertake to correct official announcements which may happen to be inaccurate. It is essential that in all cases the school of training should be given.]

Frimley Isolation Hospital.—Miss Grace Kingcott has been appointed matron. She was trained at the Kensington Infirmary, and later acted as assistant matron at the North-Western Fever Hospital, and matron of the Stone House Asylum, Canterbury.

Harton Infirmary, South Shields.—Miss Louisa Kate Clarke has been appointed night sister. She was trained at the Town's Hospital, Glasgow, for three years, and for five years held the post of charge nurse at the Union Hospital, Newcastle-on-Tyne. She holds the L.O.S. certificate.

Isolation Hospital, Herne Bay.—Miss Amelia James has been appointed nurse-in-charge. She was trained at the Bradford Corporation Hospital, and was assistant nurse and charge nurse at the Borough Fever Hospital, Dover, and charge nurse at the Blean Fever Hospital.

Port Talbot Sanatorium.—Miss Helen McInnes has been appointed matron of the Port Talbot Sanatorium, South Wales. She was trained at Fir Vale Infirmary, Sheffield, and was afterwards night nurse at the Town's Hospital, Glasgow, and has held the posts of sister of the female and maternity wards at Selly Oak Infirmary, Birmingham. She has also been engaged in private nursing in Birmingham and Glasgow, and has held the post of night sister at Stockport Union Infirmary. Miss McInnes holds the L O.S. certificate.

St. Francis Hospital for Infants, Hampstead.—Miss Katherine Thorburn has been appointed staff nurse. She was trained at the Derbyshire Royal Infirmary.

St. Leonard's Hospital, Sudbury, Suffolk.—Miss Beatrice Turner has been appointed staff nurse. She was trained at the Radcliffe Infirmary, Oxford, for three years.

Southwark Union Infirmary, East Dulwich.—Miss Isabel Kemp has been appointed second assistant matron. She was trained at the General Infirmary, Northampton, and subsequently held the posts of charge nurse at the hospital, Sutton, and head nurse and superintendent night nurse at the Southwark Union Infirmary.

Tavistock Cottage Hospital.—Miss Kate Ray has been appointed nurse-matron. She was trained at Guy's Hospital, and worked at the Fulham Fever Hospital, and later held the posts of medical sister, surgical sister, home sister, and night sister at the Seamen's Hospital, Greenwich.

### The Grace of Charity.

A recent writer points out that among the things which French people do better than English is the performance of acts of charity, which are characterised by a degree of grace and delicacy which is not common on this side of the Channel. He instances a home for girls at Sainte Agnes, founded by Mademoiselle de la Girennerie, where she and 25 companions "snatched from the turpitudes of Paris" support themselves by making suits for little boys. What strikes the English observer about this place is that "the sense of beauty is never for an instant disregarded" in the furnishing of it, though that is of necessity simple in the extreme. The girls dress as they please, and "Mademoiselle de Girennerie is careful to remember that a woman's desires in dress are an appreciable element of pleasure to her." "The shopping days are always great events." In short the place seems to be not in the least "institutional." And what a satire on our institutions is this word, which to the average mind implies the acme of dreary monotony! It is perhaps the weak point in our English charities that they show so little regard for the human instincts of their beneficiaries. It is true that a great deal depends on the kind of charity as to whether or not it should be made attractive. No one wants to make the workhouse a desirable place of residence for the able-bodied, for example, and one would never want to make the acceptance of doles agreeable; but in such a place as Sainte Agnes, where it is a question of persuading young girls who may be tempted to an evil life to prefer a good one, it is well to make allowance for tastes which in themselves are natural, but which may very easily lead a girl astray, if they are not reasonably gratified in a wholesome way. The managers of many of our "homes" for all dependent classes seem occasionally to forget that to the restraints of a home must be added its privileges, if it is to be worthy of the name. Institutions which are to have a restraining or a regenerative influence on their inmates cannot be judged simply by the excellence of the education they give or the perfection of their sanitation, if they make life a grey and dismal hing and flatten out individuality. Our consciousness cf the seriousness and strenuousness of life is one of the best features of the English character, but it is unfortunate that it should sometimes be permitted to obscure the claims of beauty and joy. Especially is this to be regretted when the serious people become philanthropists and those who crave for beauty need their help.

### Wants and Workers.

Will any reader kindly forward The Hospital a week after publication for postage prepaid? Address Miss A. L., Barr House, near Taunton.

### To Nurses.

**We invite contributions from any of our readers, and shall be glad to pay for "Notes on News from the Nursing World," or for articles describing nursing experiences, or dealing with any nursing question from an original point of view. The minimum payment for contributions is 5s., but we welcome interesting contributions of a column, or a page, in length. It may be added that notices of appointments, entertainments, presentations, and deaths are not paid for, but that we are always glad to receive them. All rejected manuscripts are returned in due course, and all payments for manuscripts used are made as early as possible after the beginning of each quarter.**

### The Nurses' Bookshelf.

Lessons on Massage. By Margaret D. Palmer. Second edition. Demy 8vo. (London: Baillière, Tindall and Cox. 1903. 7s. 6d. net.)

This is a revised edition of this excellent text-book on massage. The additions in the present volume are two useful illustrations and chapters upon the Nauheim treatment and bandaging, both necessary for the complete education of the masseuse.

"Tat." Tales and Talk. (London: Edwin J. Brett, Ltd., 6 West Harding Street, E.C. 1d. weekly.)

This new magazine professes to give a sixpenny magazine for a penny. It does not pretend to compete with the sixpenny monthlies in illustrations, though it has some neat initials, and the paper is cheap. But the type is good—which is a point worth considering for those whose eyes are not of the best, and the letterpress is genuinely interesting. There are, in this first number, short stories by Pett Ridge, Tom Gallon, and Barry Pain, and the beginning of a serial story by Fred. M. White, besides some curious information on many subjects. The shape of the magazine is long and narrow, excellently adapted for slipping into a coat pocket, and the possession of it, in case of an accident, is an insurance for £500. If "Tat" goes on as it begins, it will deserve success.

The Heart of a Great Man. By Lucy Rae. (F. V. White. 6s.)

A Secret society, a strong man, slighted beauty, and a detective are the pegs upon which Miss Lucy Rae hangs the incidents that form the plot of her novel. The book is not without promise, but the author is more at home in depicting weird, if somewhat stagey incidents, than in the lighter and less serious setting of society scenes. The opening of the first chapter leaves the reader in no uncertainty. "The Brethren of Darkness were holding a meeting." . . . "The effect of the entire assembly was sombre in the extreme. All wore black cloth masks and were dressed in the ordinary black evening clothes of private gentlemen. The table was covered with black baize, the walls were painted black, while the absence of any windows to break the monotony of straight walls gave a strangely weird and funereal appearance to the room" Nineteen members were present and opposite the President's chair a vacant place suggested an absentee; pens and paper were placed in readiness for the arrival. The particular object of the Brethren of Darkness, as a society acting for the Russian Government, was to detect schemes invented for the protection of Great Britain against foreign Powers. On this evening the subject under discussion was a plan of defence, invented by Anton Garth, a British subject and the great man of the story, which if perfected, would make the country impregnable to outsiders. Mystery now enters into the assembly. The Brethren are startled, during a momentary pause in their discussion, by a knock on the door. Number one arose, and in reply to the question, "who is without?" is answered by a "muffled voice," which declares the owner to be of the Brotherhood. The door is opened to admit a masked lady, who moves forward and takes the vacant seat. Now begins the comedy, which ends in such dire tragedy. "The Heart of a Great Man" is a book that will delight young readers who like dramatic settings to scenes which are only met with in fiction of this class, and which serve as an exciting antidote to the routine of everyday existence.

### Echoes from the Outside World.

### Movements of Royalty.

On Friday night the King returned to London, and was received at Charing Cross station with manifestations of great enthusiasm by a large crowd, which, in spite of extremely unfavourable weather, had assembled in the vicinity of the terminus. An interesting feature in connection with his Majesty's visit to Vienna was the reading of a letter by the Burgomaster of the capital before the Communal Council, from the British Ambassador, in which his Excellency stated that he had been commanded by King Edward to communicate his Majesty's warmest thanks to the city of Vienna for the brilliant and cordial welcome, which had afforded him sincere pleasure. In conclusion, the letter announced that King Edward had given 2,500 kronen (£100) to the poor of Vienna. On Monday, the King, after seeing the Queen and Princess Victoria off to Copenhagen, left London for Rufford Hall, Notts, where he will spend the week, proceeding afterwards direct to Balmoral. The Prince and Princess ef Wales and Princess Charles of Denmark are in Scotland.

### Death of the Austrian Ambassador.

The death of Count Deym, the Austro-Hungarian Ambassador to the Court of St. James's, was announced on Saturday. His Excellency was spending his leave at Erbersdorf, Neurode, in the county of Glatz, and his decease at the time when King Edward's visit to Emperor Francis Joseph had given fresh expression to the genuine friendship which exists between the two monarchs, is specially sad. Count Deym began his distinguished diplomatic career in 1860 at St. Petersburg, and was, in succession, a member of the Legations in Paris, Rome, Brussels, and London. He then for eight years sat in the Austrian Parliament, but in 1897, soon after he became a member of the Upper House, he re-entered the diplomatic service, and was appointed Ambassador in London the following year. The family he leaves behind him consists of the widow, three sons, and two daughters; but his death will also be deeply regretted by a wide circle of attached friends.

### Another Servian Plot.

Last week 47 Servian officers from different garrisons were arrested at Nish, where they had assembled to plan the murder of their brother officers connected with the assassination of King Alexander and Queen Draga. The officers implicated in this new plot had signed a violent proclamation demanding the punishment of the officers concerned in the murder of the late sovereign and his consort. The proclamation is directed specially against Colonel Popovitch and other officers of King Peter's suite, who, with his Majesty, are now staying at Nish. The movement against the assassins comprises nearly a thousand Servian officers, who have agreed to resign unless their demands are granted. Great excitement prevails at Nish, and the Minister of the Interior has returned suddenly to Belgrade to take precautions against an extension of the movement. It is doubted in the capital whether the position of King Peter will long be tenable if he continues to protect the assassins against the rest of the army.

### The Duke of Roxburghe's Engagement.

The young Duke of Roxburghe, who is only 27 years of age, is engaged to Miss May Goelet, said to be one of the richest unmarried ladies in the world. Her father died on his yacht, the *Mayflower*, four years ago. His personal estate was small when his will came to be proved, but he left to his only daughter real estate property representing several millions sterling. The Duke distinguished himself in the South African War, and risked his own life in order to save that of a private soldier. His mother was with Queen Victoria when the madman McLean fired a revolver at her, and she enjoyed the friendship of the late Sovereign over a long period of the years.

### Americans and Sir Thomas Lipton.

Following the final defeat of *Shamrock III.*, Sir Thomas Lipton was entertained on Saturday by 150 Pilgrims of the United States at a complimentary dinner in New York. The guest of the evening was loudly cheered when he rose to respond to the toast of his health. He thanked the Pilgrims and Americans generally, and the New York Yacht Club particularly, for their hospitality and courtesy, and was especially grateful for the admirable way in which the course was kept clear. The Sandy Hook course was, he said, as good as any other, indeed he did not know whether there was another in the world like it. He was still hopeful that he would see the cup on the other side, and, meanwhile, it was a consolation that conquerors and conquered were of the same good old race. It is stated that Sir Thomas has decided to sell *Shamrock I.* and *Shamrock III.* to American yachtsmen and *Shamrock II.* to a junk dealer.

### A Cornish Watering Place.

Those who like to spend their holidays by the sea, amongst beautiful hills and valleys covered with cornfields and grass lands, golden bracken, and many-hued flowers around, with bright, pure, bracing air and kindly folks to minister to their comfort, should make a note of Port Isaac. The little port itself is almost entirely devoted to fishing, and nestles on the sea between the hills. Above are farms and substantial houses and cottages scattered here and there, where visitors are welcomed and treated well, fowls and ducks, fresh butter, eggs, fruit, and Cornish cream being easy to obtain. As yet, although only 2½ miles from a station—Port Isaac Road on the London and South-Western Railway—the place is almost unknown, even to those who pride themselves upon being no strangers to the beauties of North Cornwall. But when once this picturesque corner of the county, which is close to Port Gaverne, where the bathing is good and perfectly safe, and not far from the beauties of Tintagel and Boscastle, becomes known to the world, it will probably grow into public favour as rapidly as Newquay did. At present holiday makers return year after year, and there will probably be plenty of occupants for the new houses which are springing up along the cliffs, looking over the sea with its wonderful deep blue, stretching away, landless, till it touches the American coast.

### A Woman's Club Question.

An interesting question with regard to a woman's club has arisen and will probably be settled in the Law Courts. The XXth Century Club two years ago bought the freehold of three houses in Stanley Gardens, Notting Hill. These houses, upon payment of a subscription of £2 2s. per house, are allowed to use the gardens known as Stanley Gardens and Kensington Park Gardens, and hitherto the 45 ladies resident at the club have enjoyed this privilege. Now, however, that the club is enlarging its domain by taking in three more houses the Garden Committee fear that the 100 members may take such complete possession of the gardens as to spoil the pleasure of the other residents, and the secretary of the club has been asked to forbid its members to use the enclosure. This they refused to do, and they also vetoed a second proposal of the Garden Committee that only 20 per cent. of the members should be allowed in the garden at the same time because of the difficulty of carrying out the suggestion.

### For the Guidance of Nurses.

[A Matron, seeing that a medical superintendent asks for simple directions to be hung up in typhoid wards, encloses the directions which she drew up for the help and guidance of the nurses in her hospital. There are only small wards—three beds in most of them—but it is seldom that one can be given up entirely to a typhoid case, hence the necessity for keeping all utensils strictly separate. Of course the rules require modifying for another hospital, but Matron thinks that they may perhaps be a help to our correspondent. They are pasted on cardboard and hung up over the mantelpiece in whichever ward there is a typhoid case. The feeders, etc., are marked with a red cross in Aspinall's enamel.]

### GENERAL DIRECTIONS FOR NURSING ENTERIC CASES.

1. Treat every suspected case as though it had been definitely pronounced to be enteric.

2. Mark all feeders, washing-bowls, bed-pans, urinals, etc., with a distinctive mark, and keep them separate from those used by other patients. Feeders must be washed in the special bowl provided for the purpose, and dried with the special cloth set apart for them.

3. Boil all milk and measure out the specified quantity every two hours; add the barley water or lime water ordered and see that the patient really drinks the nourishment. Do not merely place it on the locker at the bedside.

4. See that the patient has as much cold water to drink as he cares to take.

5. Keep a bowl of lotion—1 in 1,000 perchloride of mercury—on the washstand for the nurse's hands. Glycerine may be added to it if liked.

6. Keep the nails cut short. After attending to an enteric patient, brush the hands and nails and dip into the above lotion.

7. Disinfect the urine and stools with perchloride of mercury—1 in 500—and place in the typhoid pan provided. Let them stand for not less than 30 minutes in the disinfectant before being emptied down the sluice. If the stools are formed, break up with the glass rod provided for the purpose.

8. Keep the special mop in a jar of perchloride lotion—1 in 500—together with the glass rod.

9. Place all soiled bed-linen, towels, nightshirts, handkerchiefs, etc., in the bucket in the ward, and soak with carbolic lotion (1 in 20). Then take straight to the laundry and turn them into the tub of disinfectant prepared for them.

10. Never allow an enteric patient to sit up or to raise himself on his elbow until permission to do so has been given by the doctor.

11. Wash the patient all over between blankets, morning and evening, and sponge in between times as ordered by the doctor.

12. Cleanse the mouth, teeth and gums with glycerine and boracic acid twice a day at least. Keep a separate pair of forceps for the purpose.

13. Do not load the patient with bedclothes, but guard the chest, remembering that a certain amount of bronchitis generally accompanies enteric.

14. Keep the window slightly open and the room well ventilated.

15. Prepare the room for stoving as soon as the patient is discharged.

### Notes and Queries.

**REGULATIONS.**

**The Editor is always willing to answer in this column, without any fee, all reasonable questions, as soon as possible.**

**But the following rules must be carefully observed:—**

1. **Every communication must be accompanied by the name and address of the writer.**
2. **The question must always bear upon nursing, directly or indirectly.**

**If an answer is required by letter a fee of half-a-crown must be enclosed with the note containing the inquiry.**

*Surgical Instruments.*

(244) Will you kindly tell me if there is a book on surgical instruments, with names and illustrations, and mentioning those required for various operations?—*Nurse A.*

"Surgical Ward-Work and Nursing," by Dr. Alexander Miles, is the only book we are acquainted with which fulfils your requirements. It may be obtained from the Scientific Press, Limited, 28 and 29 Southampton Street, Strand, price 3s. 6d. net, 3s. 10d. post free.

*Still-born.*

(245) I am a certified midwife. Will you kindly tell me if I could give a certificate in the case of a still-born infant? Where should I apply for forms, and if I cannot give the certificate, how should I act?—*M. A. B.*

You had better ask your local Registrar of Births and deaths to instruct you as to the legal procedure.

*Oculist.*

(246) *M. V.*—The oculist you mention no longer attends at any of the London hospitals.

*Canada.*

(247) Will you kindly inform me of any home through which Scotch nurses may obtain posts in Canadian hospitals, Toronto preferred? I have relatives there who wish me to join them, but I should like to be sure of obtaining work after paying them a short visit.—*Canny Scot.*

Your relatives could best obtain the information you need, or write to the Lady Superintendent, Victorian Order of Nurses for Canada, Headquarters, 578 Somerset Street, Ottawa.

*Private Work.*

(248) Will you kindly tell me how to obtain plenty of private work? I am living in the country.—*Private Nurse.*

Advertise regularly, and extend your connection by personal recommendations from your patients and the medical men under whom you work.

*Hospital Training.*

(249) Could you tell me if at my age, 36, I am too old to enter an infirmary for three years' training (maternity objected to)? If so, is there any other way in which I can obtain training?—*I. S.*

You could advertise; as although you have passed the age limit for training, the matron of one of the smaller hospitals might be willing to train you.

I am anxious to be trained in midwifery and general nursing so as to be fitted for either district or private work. I cannot afford a high premium, and must go where I shall receive a small salary.—*E. R.*

See "The Nursing Profession: How and Where to Train." You will probably find that a workhouse infirmary, offering a course of maternity training as part of the curriculum, would suit your purpose best. Fulham Parish Infirmary, Hammersmith, W.; Lewisham Infirmary, Lewisham, S.E.; The Union Hospital, Cardiff; Croydon Union Infirmary are amongst those which offer facilities to their nurses for maternity training.

I am 24, and have had two years' asylum training and also some experience in private nursing, how can I get a certificate?—*F. H. R.*

You do not say for what kind of nursing. If for general training, it is by far the best policy to earn a three years' certificate from a well-known training school. Full particulars of the terms, salaries, and conditions of training, as well as a list of schools, are given in "The Nursing Profession: How and Where to Train."

Can you tell me what children's hospital will train me for six or twelve months? Also, if a three years' training in a children's hospital counts the same as three years in a general hospital?—*Perplexed.*

Three years' training in a children's hospital does not count the same as three years' general training. The Hospital for Sick Children, Great Ormond Street; the Belgrave Hospital for Children, Gloucester Street; and the Evelina Hospital for Sick Children, Southwark Bridge Road, all train for 12 months.

Will you kindly tell me where I can get three years'

training in general nursing in a London hospital, without a premium, and with a small salary?—*E. L.*

You might apply to the Matron at the Middlesex Hospital, Mortimer Street, W. See "The Nursing Profession: How and Where to Train," for all particulars both for this and other London hospitals.

I have a desire to train as a nurse, and would be glad if you will be so kind as to give me a little information as to how to set about it. I am 22 years of age and have good health. I have been training as a cookery teacher. It is necessary for me to have a small salary.—*O. B.*

"The Nursing Profession: How and Where to Train" will tell you exactly what you want to know.

*Menopause.*

(250) *G.* will be much obliged if the Editor will kindly tell her if there is a book on the "Menopause."

"The Menopause and its Disorders," by Dr. Leith Napier, is a standard work on the subject. It is published by the Scientific Press, Limited, 28 and 29 Southampton Street, Strand, price 7s. 6d. net, 7s. 11d. post free.

*Massage.*

(251) Can you give me particulars of Dr. Fletcher Little's Institution of Masseuses, and the address? Do you think Mrs. Creighton Hale, or the Incorporated Society, the better for training?—*K. A. C.*

The Incorporated Society of Trained Masseuses, 12 Buckingham Street, Strand, W.C., will give information concerning all courses of instruction.

*Manchester.*

(252) Will you kindly let me know if there are any private nursing homes in Manchester worked on the co-operative principle? —*Rita.*

We have no information respecting private nursing homes in Manchester.

*Naval Nurse.*

(253) Will you kindly tell me if there is such a thing as a naval nurse? Also, how one can become such, and if the knowledge of foreign languages is of any use to a hospital nurse in Great Britain or the Colonies?—*B. W. R.*

For full particulars of Queen Alexandra's Royal Naval Nursing Service apply to the Director-General, Medical Department of the Navy, Admiralty, Northumberland Avenue, W.C. A knowledge of foreign languages is always useful; but it is not essential for nursing at Home or in the Colonies.

*South Africa.*

(254) Will you give me the address of any society that supplies nurses wishing to emigrate to South Africa with posts previous to their starting. I am very anxious to get to South Africa and would be very grateful for the same.—*Nurse W.*

There is no such society; but you might be able to arrange to emigrate under the auspices of the South African Expansion Emigration Committee, 47 Victoria Street, S.W.

*Up-Country Nursing Association.*

(255) Would you kindly tell me the name and address of the Secretary of the Up-Country Nursing Association for India.—*E. W.*

Apply to Mrs. Sheppard, 10 Chester Place, Regent's Park, N.W.

*Massage.*

(256) Will you kindly inform me the right place to learn massage, the cost of the course of instruction, and the usual time it takes to get a certificate?—*K. A.*

Write to the Society of Trained Masseuses, 12 Buckingham Street, Strand, for all particulars.

*Abroad.*

(257) I am very anxious to go abroad. I have had two years at St. George's Hospital, W., and am doing staff work there. Would you kindly give me the address of an agency which would secure me work abroad?—*J. W.*

There are no nursing agencies solely for providing appointments abroad. The few associations employing English nurses on the Continent usually insist on their nurses having a three years' certificate.

### Important Nursing Textbooks.

**"The Nursing Profession: How and where to Train." 2s. net; 2s. 4d. post free.**

**"A Handbook for Nurses." (New Edition). 5s. net; 5s. 4d. post free.**

**"The Human Body." 5s. post free.**

**"Ophthalmic Nursing." (New Edition). 3s. 6d. net; 3s. 10d. post free.**

**"Gynæcological Nursing." 1s. post free.**

**"Art of Feeding the Invalid." (Popular Edition). 1s. 6d. post free.**

**"Practical Hints on District Nursing." 1s. post free.**

### For Reading to the Sick.

THE COMMUNION OF SAINTS.

Sun was shining bright and fair,
Wind was hushed, and calm was there,
Calm, and Peace beyond compare
In God's Acre.

Thought I then with joy and woe
Of dear Friends I once did know,
Sleeping now long years ago
In God's Acre.

Thought I then with throbbing brain,
Heart was beating—not with pain,
For I heard a glorious Strain
From God's Acre.

"We—the Voices seemed to say—
Far from pain and grief away,
Ever, for thee, lone One, pray,
Watches keeping.

"Ye who toil, and we that wait.
By the bright and golden Gate,
Till we reach the highest state,
In Christ are one."

Sun was shining bright and fair;
Blest Spirit-voices filled the air;
With thankful heart I said my prayer
In God's Acre.

*W. B. Flower.*

We lose much of the joy of our heavenly expectation if we do not keep in mind the Communion of Saints. Our nature expands by sympathy. There is no true joy for man but what he shares with others. It is so in time, it will be so in eternity. . . . In the Body of Christ we are all made one; and it is a unity which cannot perish, for it is knit together by the Spirit of the Lord, the Life Giver, who proceeds from Christ the Head of the Body and binds all in the glory of the Father.

Accordingly we find that Holy Scripture calls us to consider the multitude who are partakers of the same joy. The joy we shall have in Christ will be the joy of an intense sympathy with all those who delight in Him; the joy of each will be the joy of all. Oh, if we could contemplate that joy of His Redeemed, the welcome which awaits each soul as it is gathered away from earth into the blessedness of the Divine Rest; the fellowship of joy with which the blessed in Christ do even now surround the faithful who are struggling on earth, we should find the power of Christ operating towards us much more effectually than we do!—*R. M. Benson.*

If we think much of those whom we have loved on earth, and who have passed out of sight, we try to follow them; to be "imitators"—for that is the literal translation—of those who now "inherit the promises." They are above us; but not too much above us. They are still branches of the same Vine; members of the same Body. The branches of the tree are equally near to each other, whether the moonlight shine on all, or only on one branch. The hand in the shadow and the hand in the light are not more near to each other, than we are to them. If one hand is in the light, and one in the shadow, they are not really more separated, than when both were in the light, or both in the shadow. The union remains: the union with Christ, and with each other.—*Bishop Wilkinson.*

